# Genomic prediction of yield-related traits and genome-based establishment of heterotic pattern in maize hybrid breeding of Southwest China

**DOI:** 10.3389/fpls.2024.1441555

**Published:** 2024-09-09

**Authors:** Yong Xiang, Chao Xia, Lujiang Li, Rujun Wei, Tingzhao Rong, Hailan Liu, Hai Lan

**Affiliations:** Maize Research Institute/State Key Laboratory of Crop Gene Exploration and Utilization in Southwest China, Sichuan Agricultural University, Chengdu, Sichuan, China

**Keywords:** genomic prediction, maize, yield-related traits, heterotic pattern of yield, Southwest China

## Abstract

When genomic prediction is implemented in breeding maize (*Zea mays* L.), it can accelerate the breeding process and reduce cost to a large extent. In this study, 11 yield-related traits of maize were used to evaluate four genomic prediction methods including rrBLUP, HEBLP|A, RF, and LightGBM. In all the 11 traits, rrBLUP had similar predictive accuracy to HEBLP|A, and so did RF to LightGBM, but rrBLUP and HEBLP|A outperformed RF and LightGBM in 8 traits. Furthermore, genomic prediction-based heterotic pattern of yield was established based on 64620 crosses of maize in Southwest China, and the result showed that one of the parent lines of the top 5% crosses came from temp-tropic or tropic germplasm, which is highly consistent with the actual situation in breeding, and that heterotic pattern (Reid+ × Suwan+) will be a major heterotic pattern of Southwest China in the future.

## Introduction

Maize (*Zea mays* L.), one of the most important cereal crops throughout the world, has been widely used as food, biofuel, feed and raw materials of many industrial products (Yang et al., 2011a). In order to increase maize production, molecular marker techniques such as RFLP, SSR, and SNP have been developed to improve agronomic traits of economic importance (Stevens, 2008; Prasanna et al., 2010; Inghelandt et al., 2010). However, conventional marker-assisted selection (MAS) can only utilize the markers tightly linked to large or moderate-effect QTL and fails to deal with quantitative traits controlled by minor-polygene (Heffner et al., 2009; Jannink et al., 2010; Chai et al., 2012; Hao et al., 2014).

Meuwissen et al. (2001) pioneered the technique of genomic prediction to solve this problem. First, training population with genotypic and phenotypic information is utilized to compute genetic effect of each marker at the level of genome-wide markers, and then the genomic estimated breeding values (GEBV) of each individual in candidate population with only genotypic information are obtained. The fact that all marker information is included in genomic prediction contributes to higher predictive accuracy and genetic gain. Applied successfully in the breeding programs of animals and plants such as dairy cattle, beef cattle, pigs, sheep, maize, wheat, and rice (Hayes et al., 2009; Heffner et al., 2011; Meuwissen et al., 2013; Fristche-Neto et al., 2018; Cui et al., 2020; Alemu et al., 2024), many genomic prediction methods have been developed and can be categorized as (1) parametric methods such as Genomic Best Linear Unbiased Prediction (GBLUP), ridge regression Best Linear Unbiased Prediction (rrBLUP), Bayes, Haseman-Elston regression Best Linear Prediction (HEBLP), and RHPP (Meuwissen et al., 2001; VanRaden, 2008; Endelman, 2011; Habier et al., 2011; Xu et al., 2014; Liu and Chen, 2017; Liu and Yu, 2023), and (2) non-parametric methods such as Random Forest (RF), Support Vector Machine (SVM), Light Gradient Boosting Machine (LightGBM) and deep learning (DL) (Ogutu et al., 2011; Montesinos-López et al., 2018; Bellot et al., 2018; Yan et al., 2021).

Maize is the first crop to apply genomic prediction (Bernardo and Yu, 2007). Riedelsheimer et al. (2012) used 56110 SNPs to predict combining abilities of 7 biomass- and bioenergy-related traits of maize and found that prediction accuracies ranged from 0.72 to 0.81. Zhao et al. (2012) analyzed European maize elite breeding population and found that prediction accuracies of grain moisture and grain yield were respectively 0.90 and 0.58. Navarro et al. (2017) used 30000 markers to predict flower time traits including days to anthesis (DA) and days to silking (DS) for 4471 maize landraces via rrBLUP and found that the average prediction accuracy was 0.45. Li et al. (2020) utilized 8 genomic prediction methods to predict 10 traits of maize and found that the prediction accuracy ranged from 0.382 to 0.795. Xiao et al. (2021) utilized 8652 F1 hybrids from maize CUBIC population to predict days to tasseling (DTT), plant height (PH) and ear weight (EW) and found that the prediction accuracies of DTT, PH, and EW were 0.76, 0.81, and 0.66 respectively. Studies above demonstrated that genomic prediction was a highly effective technique to improve maize.

Southwest China is one of the three main production areas of maize in the country. In order to increase yield and adapt to complex ecological condition in this region, the breeders introduced tropical or subtropical maize germplasm such as ETO, Suwan, Dentado Amarillo, Tuson and Tuxpeno into the local maize germplasm to broaden the narrow genetic base (Zhang et al., 2016; Leng et al., 2019). Furthermore, a number of inbred lines containing tropical or subtropical maize germplasm were used to obtain excellent hybrid. In this study, we generated 2077 hybrids derived from random crosses of 360 inbred lines to perform genomic prediction analysis of 11 yield-related traits and furthermore established genomic prediction-based heterotic pattern of yield.

## Materials and methods

### Phenotypic data collection of maize

In this study, 360 maize inbred lines of wide genetic background and origin were collected, including the most representative inbred lines in Southwest China, inbred lines exchanged from the other breeding units of Sichuan, temperate inbred lines introduced from North China, tropic inbred lines introduced from the other breeding units, and inbred lines cultivated by our institute (**Supplementary Table S1**). From 64,620 crosses obtained through pairing of the 360 inbred lines, 2077 were randomly selected to perform field evaluation. All hybrids were planted in Field data of 11 traits including row number per ear, ear length, ear diameter, grain number per row, grain length, grain width, grain thickness, hundred grain weight, weight per unit volume, and yield (kg/mu) for 2077 crosses were collected at Gasa town, Jinghong city (21°95^’^N, 100°75^’^E, 520 meters above sea level), Yunnan province, China, in 2019. All hybrids were planted in one-row plot using a complete block design. Ten plants were planted in each row. The row length was 2.5 m and row spacing was 0.8 m. The plant density was about 3300 plants/mu. Field management followed standard procedures. For quality control, five plants in the middle of each row were selected to obtain the phenotypes. No further phenotypic corrections were performed on the basis of the other hybrid.

### Genotypic data collection of maize inbred lines

Genomic DNA of the 360 inbred lines were extracted via CTAB method, and their qualities were evaluated with a Nanodrop 2000 Spectrophotometer (Thermo Fisher Scientific). High-quality DNA was genotyped via 48K liquid phase gene chip and sequenced on an Illumina Nova 150-bp paired-end sequencing platform in China Golden Marker (Beijing, China). Quality control of raw reads was performed via Trimmomatic-0.36 (Lohse et al., 2012) with default parameter, and then the filtered data were mapped to B73_RefGen_v4 genome with BWA software (Li and Durbin, 2009). The SNPs were called with GATK (McKenna et al., 2010) and further filtered with VCFtools (Danecek et al., 2011). The parameters used in SNP filters ration were: –minDP 4, –minGQ 10, –max-missing 0.8, –maf 0.05.

### Construction of phylogenetic tree

45425 SNPs were used to generate distance matrix using VCF2Dis (https://github.com/BGI-shenzhen/VCF2Dis) and then phylogenetic tree was constructed based on distance matrix via FastME2.1.6.4 (Lefort et al., 2015).

### Genotyping of maize hybrids

The genotypes of 2077 hybrid were obtained based on those of the inbred lines. 33009 of 45425 SNPs were kept according to the following criteria: (1) there are at least two kinds of genotype at each locus; (2) the percentage of “NA” at each locus is not more than 0.3; (3) the percentage of rare alleles at each locus is not less than 0.1.

### Estimation of SNP heritability

The genetic model of a quantitative trait can be written as


y=Xb+Zu+e

where *y* is an n×1 vector for the phenotypic values; *X* is a n×p incidence matrix for fixed effects; *b* is a p×1 vector of fixed effects; *p* is the number of fixed effects; *Z* is n×m genotype matrix; *m* is the number of markers; *u* is a m×1 vector of additive effects following $N\left(0,I\sigma_{u}^{2}\right)$ ; *e* is a n×1 vector of residual error following $N\left(0,I\sigma_{e}^{2}\right)$ . The SNP heritability was calculated as:


$$h^{2}=\frac{\sigma_{g}^{2}}{\sigma_{g}^{2}+\sigma_{e}^{2}}$$

We estimated $\sigma_{g}^{2}$ and $\sigma_{e}^{2}$ via the program GCTA v1.94.1, which consists of two steps: (1) computing the genetic relationship matrix (GRM) of all individuals based on all SNPs; (2) estimating additive genetic variance of a trait based on above GRM via the restricted maximum likelihood (REML) (Yang et al., 2011b).

### Evaluation of genomic prediction of 11 yield-related traits

Two parametric genomic prediction methods including rrBLUP (Endelman, 2011) and HEBLP|A (Liu and Chen, 2017) and two non-parametric genomic prediction methods including RF and LightGBM were used in this study.

rrBLUP is one of the first methods for genomic prediction and has been taken as a baseline model (Meuwissen et al., 2001). The formula of rrBLUP is written as the following:


y=μ+Zu+e

in which y is the vector of the phenotype; μ is the vector of mean value; Z is the genotype matrix; u is the vector of additive effects of the causal loci following $\text{N}\left(0,{\text{Iσ}}_{\text{u}}^{2}\right)$ where $\sigma_{\text{u}}^{2}$ is the additive genetic variance; e is the vector of residual error following $\text{N}\left(0,{\text{Iσ}}_{\text{e}}^{2}\right)$ . The BLUP solution for u is written as $\hat{\text{u}}={\text{Z}}^{\text{T}}{\left({\text{ZZ}}^{\text{T}}+\text{λI}\right)}^{-1}\left(\text{y}-\hat{\text{μ}}\right)$ where $\text{λ}=\frac{\sigma_{\text{e}}^{2}}{\sigma_{\text{u}}^{2}}$ . The ridge parameter λ is computed on the tenfold cross-validated partial likelihood that minimized the residuals between predicted and observed phenotypes within the training population.

HEBLP|A is highly efficient in computation due to its estimation of heritability via Haseman-Elston regression (Liu and Chen, 2017). HEBLP|A and rrBLUP have similar model, but are different in the strategy of computing λ. HEBLP|A uses IBS-based Haseman-Elston (HE) to estimate $\sigma_{\text{u}}^{2}$ and then $\text{λ}=\frac{1-\sigma_{\text{u}}^{2}}{\sigma_{\text{u}}^{2}}$ .

RF is one of the most popular and powerful machine learning algorithms and has already been applied to a wide variety of genomic problems (Ogutu et al., 2011). RF uses bootstrap sample of the training population to build B decision trees and then averages the output of the ensemble of B trees to predict candidate individuals. The formula is as follows:


$${\hat{\text{f}}}^{\text{B}}\left(\text{x}\right)=\frac{1}{\text{B}}\sum_{\text{i}=1}^{\text{B}}\text{T}\left(\text{x},{\text{ψ}}_{\text{i}}\right)$$

in which ${\text{ψ}}_{\text{i}}$ is the ith RF trees.

LightGBM, a very popular machine learning algorithm, was developed by Microsoft in 2017 and has been widely used in dealing with extremely large data with ultra-high efficiency (Ke et al., 2017). LightGBM uses a gradient-based one-side sampling (GOSS) and exclusive feature bundling (EFB) to reduce computational time. The basic process of the LightGBM is described as follows (Mienye and Sun, 2022):

1. Merge mutually exclusive features x_i_ based on training population $\text{S}=\left({\text{x}}_{\text{i}},{\text{y}}_{\text{i}}\right)$ using the EFB.
2. Initialize ${\text{Θ}}_{0}\left(\text{x}\right)={\text{argmin}}_{\text{c}}\sum_{\text{i}}^{\text{n}}\text{L}\left({\text{y}}_{\text{i}},\text{c}\right)$ where L(y,c) is the loss function.
3. For t=1,…,T:

  - Compute the absolute values of gradients:

${\text{r}}_{\text{i}}={\left[\frac{\text{δL}\left({\text{y}}_{\text{i}},\text{Θ}\left({\text{x}}_{\text{i}}\right)\right)}{\text{δΘ}\left({\text{x}}_{\text{i}}\right)}\right]}_{\text{Θ}\left(\text{x}\right)={\text{Θ}}_{\text{t}-1}\left(\text{x}\right)}$

  - Resample the training population using the GOSS:

D=A+B where A is samples with larger gradients and B is samples with small gradients.
  - Calculate information gain values.
  - Obtain a new decision tree ${\text{Θ}}_{t}{\left(\text{x}\right)}^{'}$ on D.
  - Update ${\text{Θ}}_{\text{t}}\left(\text{x}\right)={\text{Θ}}_{\text{t}-1}\left(\text{x}\right)+{\text{Θ}}_{\text{t}}{\left(\text{x}\right)}^{,}$ .
4. Output: $\hat{\text{θ}}\left(\text{x}\right)={\text{Θ}}_{\text{T}}\left(\text{x}\right)$ .

For RF and LightGBM methods, we utilized “randomForest” and “LightGBM” function in R package with the default parameters. The correlation coefficient between the phenotypes and the predicted genotypic values was considered as the prediction accuracy. 10 replications were used to evaluate the prediction accuracy of genomic prediction methods.

### Genomic prediction of yield of all crossing combinations

The 360 inbred lines generated 64620 crossing combinations in total. We utilized 2077 hybrids with the phenotypic and genotypic data to predict the yield performance of 64620 crossing combinations via HEBLP|A under additive model.

## Results

### Phylogenetic tree analysis of the 360 maize inbred lines

**Figure 1:**
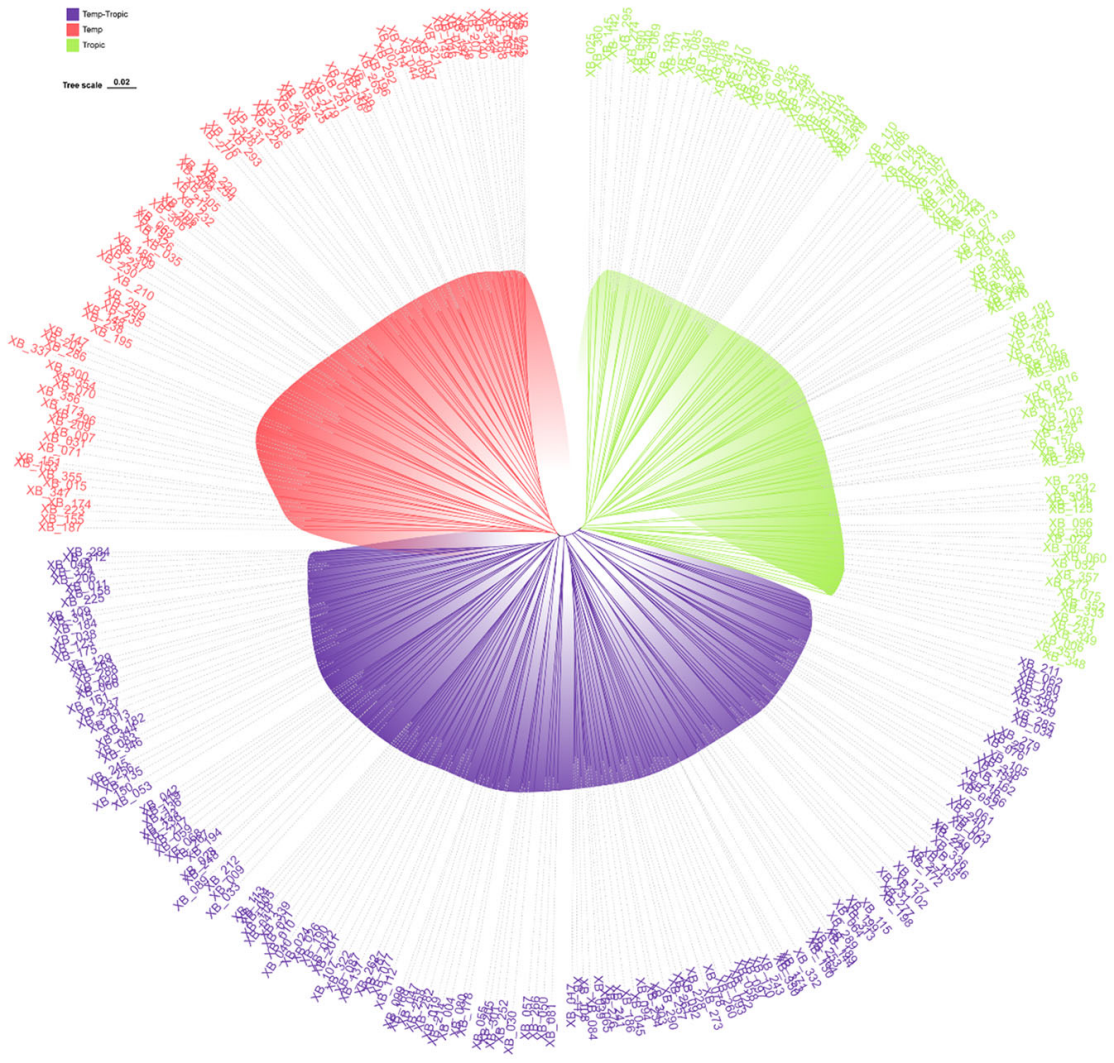
Phylogenetic tree for 360 maize inbred lines using 45425 SNP markers. Temperate germplasm (red), Temp-Tropic germplasm (purple), and Tropic germplasm (green).

As was demonstrated in the phylogenetic tree, the 360 maize inbred lines fell into three groups including temperate germplasm (90 inbred lines), tropical germplasm (106 inbred lines), and temp-tropic germplasm (164 inbred lines) (**Figure 1**). The temperate group contains temperate inbred lines from North China such as Zheng58, Chang7-2, Mo17, Dan340, PH6WC, PH4CV, and Jing724 and those from Southwest China such as Y9614 and 4011. Most of the inbred lines in the tropical group have mixed genetic background of Suwan, Tuxpeno, and CIMMTY, with the parent lines of main hybrids in Southwest China QR723, WG646, ZNC442, Xian21A, Rekangbai67, XZ50612, and 7031 being examples. As for temp-tropic group, most inbred lines such as SCML0849, CL11, R18, GH35, F19, SH1070, Q1 come from PB inbred lines and improved Reid inbred lines.

### Evaluation of genomic prediction for the 11 yield-related traits in maize hybrids

**Figure 2:**
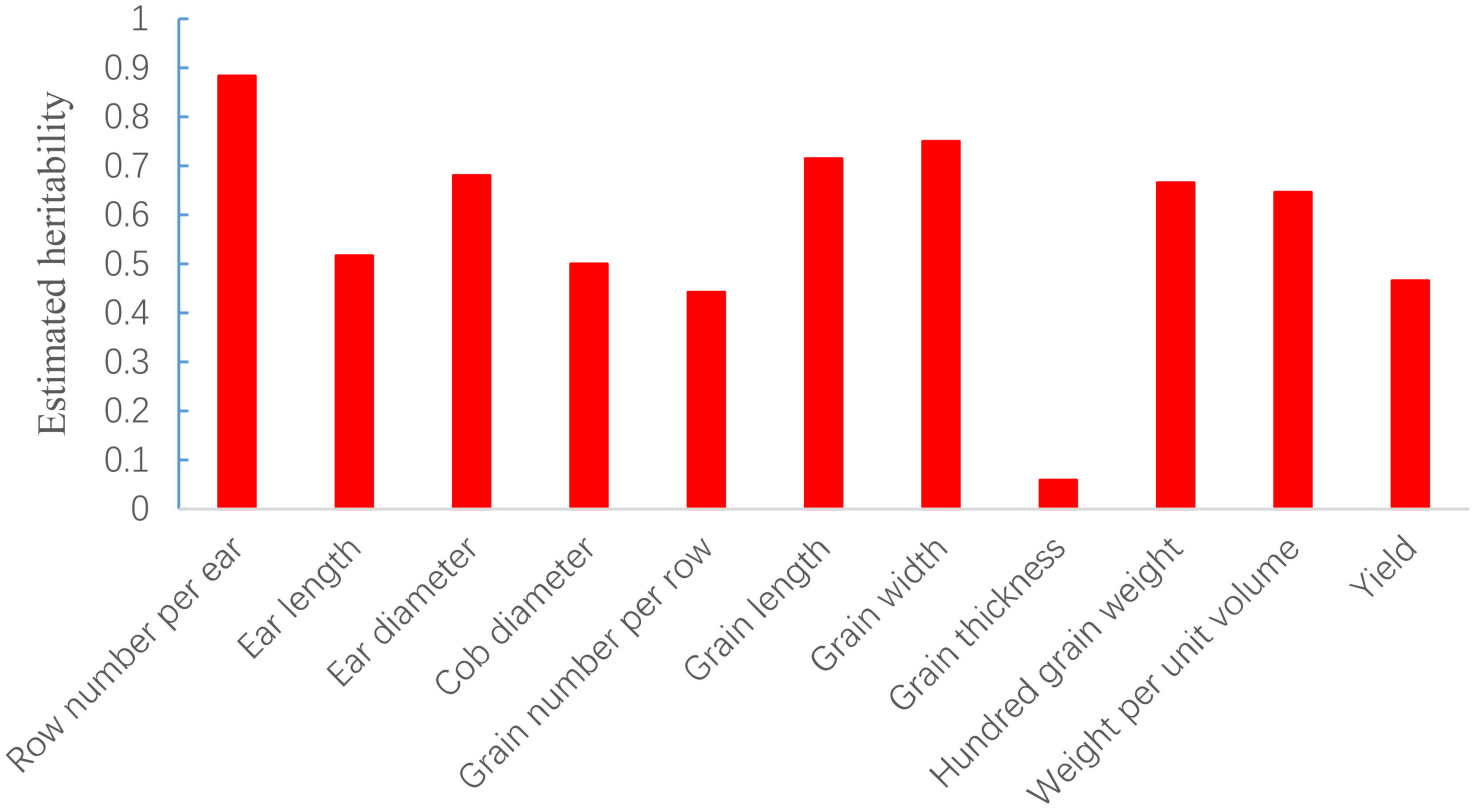
SNP-based heritability estimation of 11 traits in maize.

The SNP heritability of the 11 yield-related traits of 2077 hybrids was estimated via GCTA analysis under the additive model and ranged from 0.058 for grain thickness to 0.883 for row number per ear (**Figure 2**). The result indicated that most traits were mainly controlled by additive effects.

Evaluation of genomic prediction for the 11 yield-related traits was performed via rrBLUP, HEBLP|A, RF, and LightGBM (**Table 1**) with the size of training population and candidate population being 1800 and 277 respectively. The prediction accuracies ranged from 0.232 to 0.833 across traits and models. The row number per ear showed the highest prediction accuracy (average value across all methods being 0.796) and grain thickness had the lowest prediction accuracy (average value cross all methods being 0.302). The Pearson’s correlation coefficient between heritability and average prediction accuracy was significant ( R=0.929 and P<0.001 ), indicating that the heritability influenced prediction accuracy to a great extent, and the result was consistent with previous researches (Daetwyler et al., 2008; Liu and Chen, 2018). Among the four methods, rrBLUP and HEBLP|A have similar prediction accuracy in all the 11 traits (for example, ${\text{r}}_{\text{rrBLUP}}=0.833\pm 0.007$ and ${\text{r}}_{\text{HEBLP}|\text{A}}=0.825\pm 0.007$ in row number per ear), and so do RF and LightGBM (for example, ${\text{r}}_{\text{RF}}=0.752\pm 0.008$ and ${\text{r}}_{\text{LightGBM}}=0.772\pm 0.008$ in row number per ear). The parametric methods (rrBLUP and HEBLP|A) outperformed non-parametric methods (RF and LightGBM) in 8 traits (Row number per ear, Ear length, Ear diameter, Grain number per row, Grain length, Grain width, Hundred grain weight, and Weight per unit volume), and the latter outperformed the former only in grain thickness, which may be mainly controlled by non-additive effects such as dominance, epistasis and genotype-by-environment interaction. Both two categories of methods had similar predictive accuracy in 2 traits (Cob diameter and Yield).

**Table 1:** Comparison of genomic predictability among rrBLUP, HEBLP|A, RF, and LightGBM for 11 yield related traits of maize based on 10 simulations.

Trait	Training	Candidate	rrBLUP	HEBLP|A	RF	LightGBM
Row number per ear	1800	277	0.833±0.007	0.825±0.007	0.752±0.008	0.772±0.008
Ear length	1800	277	0.627±0.026	0.628±0.025	0.584±0.020	0.585±0.024
Ear diameter	1800	277	0.749±0.004	0.727±0.006	0.693±0.007	0.712±0.006
Cob diameter	1800	277	0.679±0.013	0.653±0.016	0.641±0.010	0.650±0.010
Grain number per row	1800	277	0.561±0.012	0.561±0.013	0.509±0.020	0.495±0.015
Grain length	1800	277	0.677±0.009	0.670±0.009	0.599±0.010	0.613±0.010
Grain width	1800	277	0.767±0.009	0.769±0.009	0.675±0.009	0.706±0.007
Grain thickness	1800	277	0.250±0.045	0.232±0.042	0.382±0.055	0.342±0.062
Hundred grain weight	1800	277	0.742±0.011	0.726±0.013	0.649±0.06	0.683±0.010
Weight per unit volume	1800	276	0.783±0.008	0.763±0.009	0.714±0.012	0.747±0.007
Yield	1800	264	0.680±0.006	0.653±0.007	0.650±0.012	0.650±0.007

The correlation coefficient between the phenotypes and the predicted genotypic values was considered as the prediction accuracy. The values after ± represent the corresponding standard error.

### Prediction of GEBVs of yield for all crossing combinations

GEBVs for yield of the 64620 crosses generated by the 360 inbred lines were obtained via 2077 hybrids with phenotypic and genotypic values under additive model via HEBLP|A and were sorted in descending order. The average GEBV of all crosses was 341.50 for yield, and that of the top 200 crosses, the top 5% (3231 ones) and the top 10% (6462 ones) was 558.35, 491.78 and 469.20 respectively. If breeders use the crosses of the best predictive accuracy in cultivation (the top 10% for example), a significant genetic gain (469.20−341.50=127.7) can be expected.

**Figure 3:**
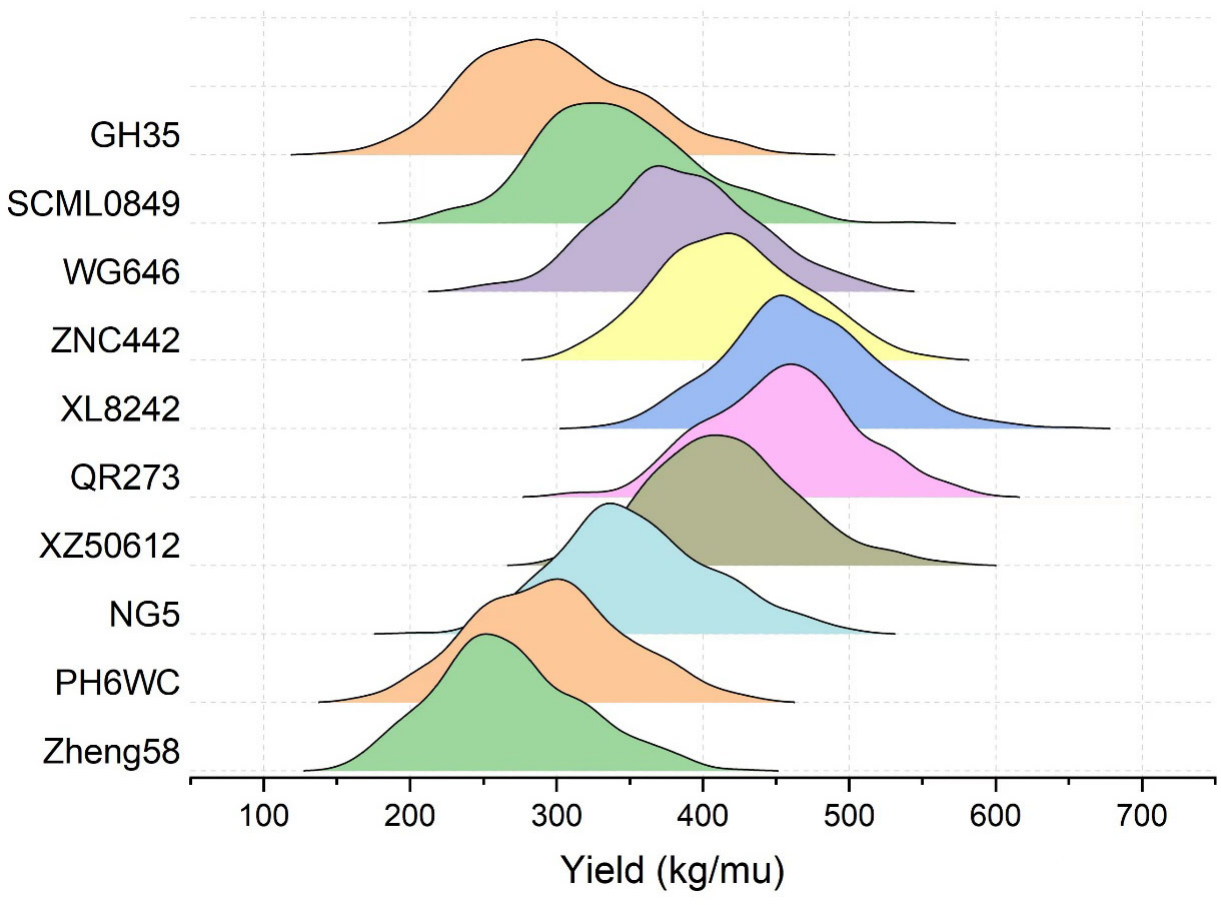
The GEBV distribution of the 359 crosses corresponding the representative inbred lines in Southwest China. Zheng58 (temperate line from North China) was taken as control inbred line. Temperate inbred line in Southwest China (PH6WC), temp-tropic inbred lines in Southwest China (GH35, SCML0849, and NG5), and tropic inbred lines in Southwest China (WG646, ZNC442, XZ50612, QR273, and XL8242).

To evaluate the general combining ability (GCA) of an inbred line, its mean GEBV of the 359 crosses was obtained by crossing the remaining 359 ones with it, and consequently there were 44 inbred lines with mean GEBVs over 400kg, among which some most popular inbred lines in Southwest China were found, such as ZNC442, QR273, YA8201, Xian21A, Rekangbai67, XZ50612, and F19. There are 29 tropic inbred lines (proportion is 65.9%) and 11 temp-tropic ones (proportion is 25%) among the 44 inbred lines, indicating that inbred lines with tropic and temp-tropic germplasm play an important role in the maize hybrid breeding of Southwest China. In contrast, the mean GEBVs of some pure temperate inbred lines introduced from North China such as 478, Zheng58, Chang7-2, Dan340, 91227, M54, and NH60 were quite low, but their improved version demonstrated high GCA when tropic germplasm was added. For example, comparing the inbred line 91227 with LX2715, its improved version via introducing tropic germplasm, we found a significant increase of the mean GEBV from 297.2kg to 349.3kg. Therefore, tropic or temp-tropic germplasms can help pure temperate inbred lines from North China to adapt to ecological environment, reducing their defects in Southwest China such as low resistance to diseases and pests when they are used in maize breeding. Generally speaking, most of the local temperate inbred lines from Southwest China contain a certain proportion of tropic germplasm through long-term improvement. In addition, the result of some representative inbred lines in Southwest China including PH6WC (temperate), GH35, SCML0849, and NG5 (temp-tropic), and WG646, ZNC442, XZ50612, QR273, and XL8242 (tropic) has showed that tropic inbred lines have better performance than temperate and temp-tropic ones (**Figure 3**). In particular, the mean GEBV of the 359 crosses corresponding to XL8242 (a newest tropic inbred line cultivated by our unit) ranked the first among the 360 inbred lines and has been used to breed some hybrids successfully (**Table 2**).

**Table 2:** Basic information of the maize varieties from 5% top crosses based on genomic prediction of yield.

Predictive rank	Name of variety	Approval number	Male parent	Female parent	Heterotic pattern
184	Youdi899	National test20231073	SCML0849	XL8242	Tropic × Temp-Tropic
249	Chuanqing9	Sichuan test20222061	LX2715	XL8242	Tropic × Temp-Tropic
1294	Yayu68	Yunnan test201105	F06	YA8201	Tropic × Temp-Tropic
1352	Rongyuqingzhu1	National test20180178	SCML5409	Xian21A	Tropic × Temp-Tropic
1752	Shidi6	Yunnan test2022102	Y9614	XB_320	Tropic × Temp
2010	Qixiangqingzhu6	Yunnan test2023161	XB_267	XL8242	Tropic × Temp-Tropic
2139	Rongkefengzan	Yunnan test2022086	NG0715	XL8242	Tropic × Temp
2255	Nonghua606	Guangxi test2017004	Y0921	ZNC442	Tropic × Temp-Tropic
2520	Chuandan99	National test20210096	ZNC442	SCML0849	Tropic × Temp-Tropic
2525	Ronghe99	National test20220514	SCML0849	XL2142	Tropic × Temp-Tropic

**Figure 4:**
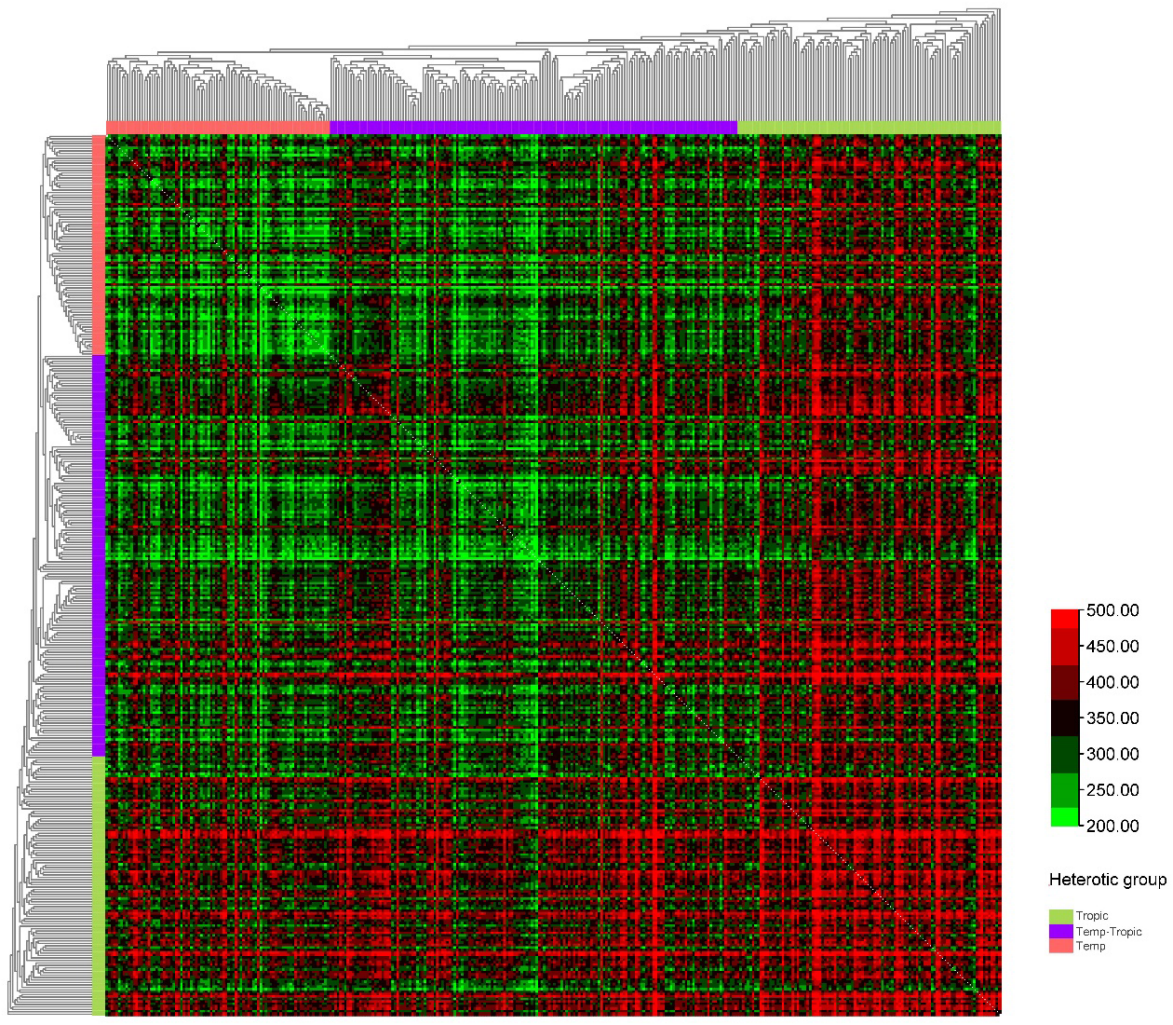
Heterotic pattern in maize hybrid breeding of Southwest China based on genomic prediction of yield (kg/mu).

**Figure 5:**
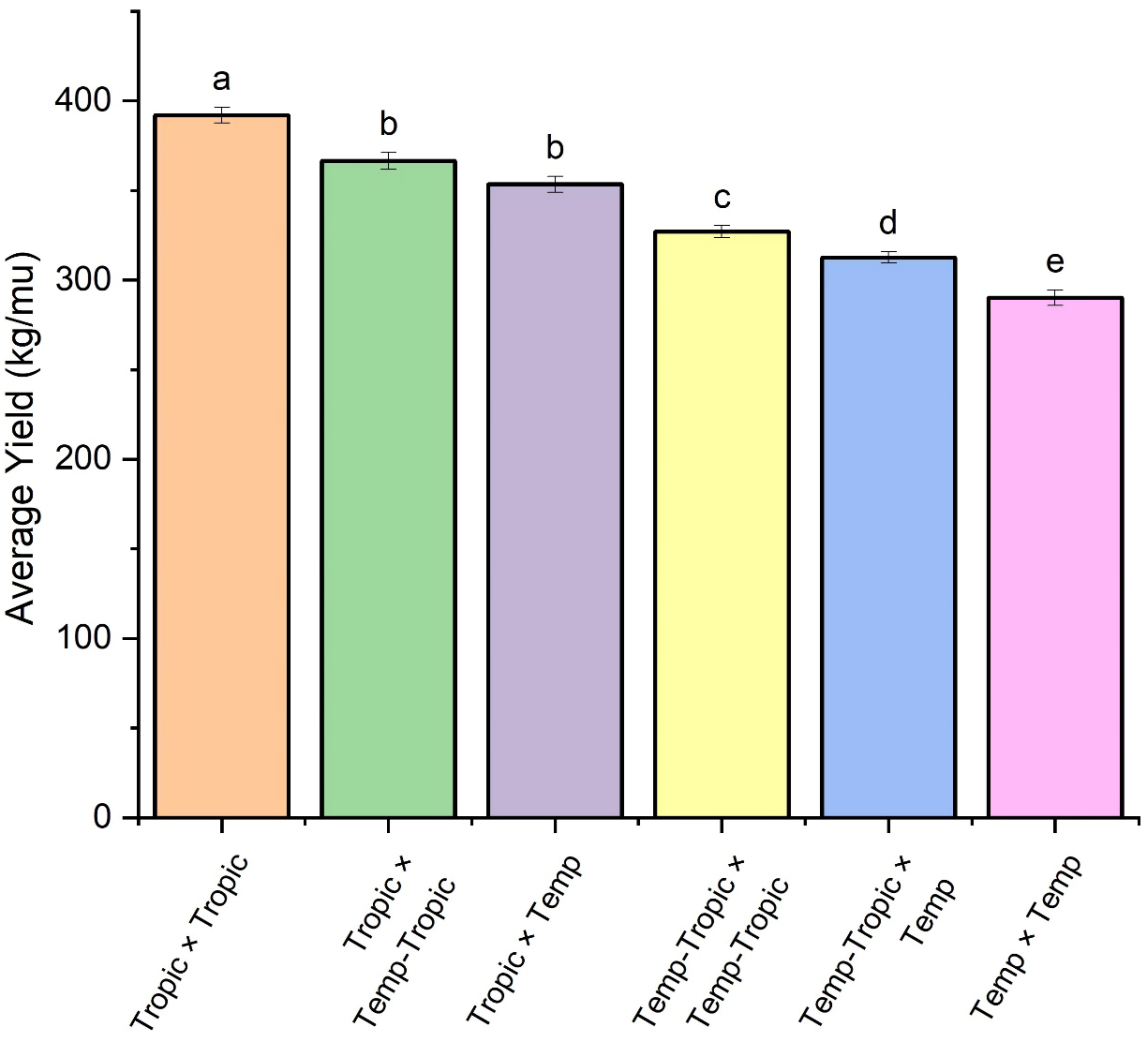
Average yield of different heterotic groups based on genomic prediction. The lowercase letters (a, b, c, d, and e) have significant difference.

**Figure 6:**
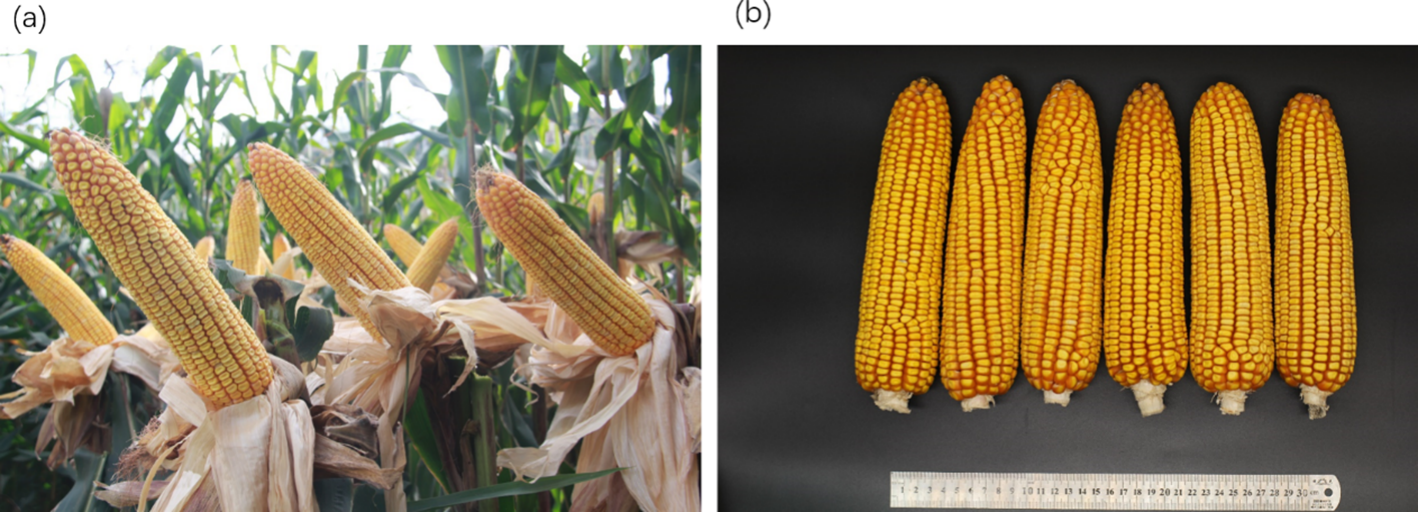
Phenotype of maize variety Youdi899. **(A)** Plant architecture. **(B)** Ear.

We constructed six heterotic patterns with three germplasms (**Figure 4**) and the results showed that the mean GEBV of Tropic × Tropic, Tropic × Temp-Tropic, Tropic × Temp, Temp-Tropic × Temp-Tropic, Temp-Tropic × Temp and Temp × Temp is 391.9±45.0 , 366.4±46.5 , 353.4±45.6 , 327.0±42.2 , 312.6±41.8 , and 290.1±41.6 respectively (**Figure 5**). Among the 5% top crosses, 21% are Tropic × Tropic mode and 66.4% are Temperate (or Temp-Tropic) × Tropic mode, indicating that Temperate (or Temp-Tropic) × Tropic mode is a major heterotic pattern. There are some approved commercial varieties among the 5% top crosses including Youdi899, Chuanqing9, Yayu68, Rongyuqingzhu1, Shidi6, Qixiangqingzhu6, Rongkefengzan, Nonghua606, Chuandan99, and Ronghe99 (**Table 2**). Take Youdi899 for example, deriving from SCML0849 and XL8242, it ranked 184 in all crosses and had an excellent performance in Southwest China (**Figure 6**). In 2020-2022 years’ national spring maize cultivar regional trials (middle and high altitude region) in Southwest China, its mean yield of two years is 814.3kg and the production increased by 14.8% compared with the control variety. In 2021-2022 years’ autonomous production test, its mean yield was 783.1kg and the production increased by 11.6% compared with the control variety. After the identification of field inoculation diseases, this variety was highly resistant to northern maize leaf blight and southern maize leaf blight, moderately resistant to ear rot, maize stalk rot, grey leaf spot, southern maize rust, and susceptible to northern maize leaf blight. Tropic × Tropic mode account for relative high proportion because Jinghong City of Yunnan Province, the place where the training population of maize was planted, belongs to tropic-subtropic region, and the result conforms well to practical breeding. In the future, we will further investigate heterotic mode of maize in other regions of Southwest China (including middle and low altitude region and middle and high altitude region) via genomic prediction.

## Discussion

It is critical for modern plant breeding to predict superior individuals with high accuracy (Heffner et al., 2009; Voss-Fels et al., 2019). When there is a large number of inbred lines in the process of maize breeding, it is difficult to identify superior hybrid crosses from $\frac{N*\left(N-1\right)}{2}$ crosses (*N* represents number of inbred lines) via conventional breeding methods because it is time consuming and too expensive. Being a useful technique to select superior hybrid crosses, genomic prediction has been successfully applied to maize breeding currently (Albrecht et al., 2011; Zhao et al., 2012; Riedelsheimer et al., 2012; Technow et al., 2014; Li et al., 2020; Wang et al., 2020; Luo et al., 2023).

To our knowledge, this is the first study about a large-scale application of genomic prediction in Southwest China’s maize hybrid breeding. We evaluated the genomic prediction accuracies of 11 yield-related traits in an F1 population with 2077 F1 maize hybrids and found that the predictive accuracies of most traits except grain thickness were higher than 55%. It is indicated that genomic prediction is a promising tool in maize breeding programs. Comparison of four methods demonstrated that parametric ones (rrBLUP and HEBLP|A) outperformed non-parametric ones (RF and LightGBM) in 8 out of 11 traits, which is consistent with previous studies that parametric methods outperformed machine-learning methods when the traits were mainly controlled by additive effects (Abdollahi-Arpanahi et al., 2020; Alves et al., 2020; Yu et al., 2023).

Increasing yield is the most important objective in hybrid maize production (Li et al., 2011; Peng et al., 2011; Tian et al., 2024), but it is difficult to improve yield trait via phenotypic selection or conventional MAS because it is a complex trait controlled by a large number of quantitative trait loci (QTL) with small effects (Ndlovu et al., 2022). In this study, the predictive accuracy of yield was about 65% when four genomic prediction methods were evaluated with 2077 maize crosses. According to the prediction of the yield performance of all 64620 crosses, one parent of the top 5% crosses has at least a tropic or temp-tropic inbred lines, and 86.4% of those top 5% have at least a tropic inbred line, which is highly consistent with practical breeding. It is indicated that tropic inbred lines play an important role in selecting superior hybrids in Southwest China.

With serious increase of diseases and pests of maize at present, it is more and more difficult for Reid × Suwan, the conventional heterotic pattern of Southwest China (Ni et al., 1996; Pan et al., 2020) to tackle the problem. Therefore, we proposed a new heterotic pattern (Reid+ × Suwan+) in which Reid+ represents the introgression from tropical germplasm or other temperate germplasm to Reid inbred lines, and Suwan+ represents the introgression from other tropical germplasm to Suwan inbred lines. It will become a major heterotic pattern of Southwest China in the future.

## Data Availability

The original contributions presented in the study are included in the article. Further inquiries can be directed to the corresponding authors.
